# Problematic assumptions have slowed down depression research: why symptoms, not syndromes are the way forward

**DOI:** 10.3389/fpsyg.2015.00309

**Published:** 2015-03-23

**Authors:** Eiko I. Fried

**Affiliations:** Research Group of Quantitative Psychology and Individual Differences, Faculty of Psychology and Educational Sciences, University of LeuvenLeuven, Belgium

**Keywords:** DSM, depression symptoms, essentialism, major depression, networks, nosology

## Abstract

Major depression (MD) is a highly heterogeneous diagnostic category. Diverse symptoms such as sad mood, anhedonia, and fatigue are routinely added to an unweighted sum-score, and cutoffs are used to distinguish between depressed participants and healthy controls. Researchers then investigate outcome variables like MD risk factors, biomarkers, and treatment response in such samples. These practices presuppose that (1) depression is a discrete condition, and that (2) symptoms are interchangeable indicators of this latent disorder. Here I review these two assumptions, elucidate their historical roots, show how deeply engrained they are in psychological and psychiatric research, and document that they contrast with evidence. Depression is not a consistent syndrome with clearly demarcated boundaries, and depression symptoms are not interchangeable indicators of an underlying disorder. Current research practices lump individuals with very different problems into one category, which has contributed to the remarkably slow progress in key research domains such as the development of efficacious antidepressants or the identification of biomarkers for depression. The recently proposed network framework offers an alternative to the problematic assumptions. MD is not understood as a distinct condition, but as heterogeneous symptom cluster that substantially overlaps with other syndromes such as anxiety disorders. MD is not framed as an underlying disease with a number of equivalent indicators, but as a network of symptoms that have direct causal influence on each other: insomnia can cause fatigue which then triggers concentration and psychomotor problems. This approach offers new opportunities for constructing an empirically based classification system and has broad implications for future research.

## Introduction

Major depression (MD) is a highly prevalent, impairing, recurrent, and often chronic disorder (Solomon et al., 2000; Kessler et al., 2003, 2005; McClintock et al., 2010), and one of the most pressing health-related problems of modern living. Despite decades of research, however, very basic questions remain unresolved: genetic studies have been unable to identify loci reliably associated with depression diagnosis (Lewis et al., 2010; Shi et al., 2011; Wray et al., 2012; Daly et al., 2013) or treatment response (Tansey et al., 2012), antidepressants do not work above placebo level for the majority of patients (Khan et al., 2002; Kirsch et al., 2008; Pigott et al., 2010), and the recently published DSM-5 field trials have documented a “questionable” reliability for depression diagnosis (Regier et al., 2013).

One of the main reasons for this striking lack of progress is *covert heterogeneity* of depression: the current diagnostic criteria and common research practices lump individuals suffering from diverse psychiatric symptoms into one undifferentiated category (Olbert et al., 2014; Zimmerman et al., 2014). To qualify for a diagnosis of MD, individuals have to exhibit 5 or more criterion symptoms, at least 1 of which has to be either ‘sad mood’ or ‘loss of interest.’ A recent study identified 1030 unique depression symptom profiles in 3703 individuals diagnosed with MD, translating into only 3.6 patients per profile (Fried and Nesse, 2015). The most common profile had a frequency of only 1.8 and 41.5% of the participants endorsed symptoms patterns that were present only five times in the full sample. It has also been shown that depression symptoms such as insomnia, fatigue, sad mood, or concentration problems differ from each other in important aspects. For instance, symptoms differ in their risk factors (Lux and Kendler, 2010; Fried et al., 2014) and underlying biology (Hasler et al., 2004; Myung et al., 2012; Kendler et al., 2013), exhibit variable impacts on impairment of psychosocial functioning (Tweed, 1993; Faravelli et al., 1996; Fried and Nesse, 2014), and particular adverse life events trigger specific symptom profiles (Keller and Nesse, 2006; Keller et al., 2007).

This means that sum-scores obfuscate important differences between symptoms on the one hand, and between individuals on other hand. In this paper I aim to explain why sum-scores are so prevalent in depression research, and elucidate two main assumptions they are tacitly based on. I go on to show that these assumptions contrast with evidence, and conclude with discussing an alternative network framework that accommodates both the fuzzy nature of MD as well as the stark differences between individual symptoms better than the current perspective.

## The Current Depression Schema

Why is information about specific symptoms commonly disregarded in favor of unspecific sum-scores and diagnoses? Two implicit assumptions of the currently dominating research framework have encouraged the pervasive use of sum-scores. It is important to point out that few researchers and even fewer clinicians will defend these assumptions. In a sense, we as a community conduct research based on these assumptions, while most individual researchers may not hold them. The goal here is not to attack a straw man, but to explicate these problematic assumptions, and elucidate how deeply entrenched they are in everyday research practices. Not only are they are reflected in how we think about MD, but also in how we pose research questions, and in the statistical models we use to answer such questions. Globally, the assumptions have fostered and continue to foster simplistic thinking about depression, and have greatly contributed to the disappointingly slow progress in key research areas.

### Depression as Distinct Disease Category

The first assumption is that depression constitutes a distinct disease category, similar to medical conditions such as pneumonia or myocardial infarction. Historically, the view of diseases as specific entities was solidified by the discovery of causative agents for infectious diseases. In 1905, the German microbiologist Robert Koch won the Nobel Prize for identifying the organisms that cause infectious diseases like anthrax and tuberculosis. The subsequent discovery of specific bacteria causing other specific diseases, such as *Treponema pallidum* for syphilis, consolidated the understanding of medical disorders as natural kinds (Boyd, 1999; Zachar and Kendler, 2007; Kendler et al., 2010). This perspective views diseases as unchanging and ahistorical entities with sharp boundaries accounted for by specific causes. Diseases are defined by a specific set of properties (e.g., symptoms and duration) that are both necessary and sufficient for a diagnosis. This particular way of classification is often referred to as *essentialism* or *kind essentialism* (Wilson et al., 2007, p. 3; Kendler et al., 2010), and an essence in this sense can be defined as “some kind of underlying, intrinsic property, something that lies within kind members, making them the kind of thing that they are.” All members of a kind have certain intrinsic properties, and identifying these properties allows for a reliable classification.

Chemical elements provide good examples for natural kinds: gold has the atomic number 79, and everything with this atomic number is gold. The internal structure itself defines kind membership, not a man-made classification system. Measles, on the other hand, is an infection of the respiratory system caused by a specific virus, and accompanied by various symptoms like red eyes, fever, and a generalized rash. Moreover, many individuals suffering from measles exhibit a pathognomonic symptom – Koplik’s spots inside the patient’s mouth – that allows for a diagnosis beyond any reasonable doubt.

This disease model has been considered one of the most important discoveries in medicine (Hyland, 2011), and has been crucial in the development of efficacious treatments. The successful treatment of tuberculosis requires at least three insights: that tuberculosis is caused by a specific bacterial agent; that the underlying disease causes particular symptoms, which in turn indicate the presence of the latent disorder; and that antibiotics are successful in the treatment of such a bacterial infection.

The idea of diseases as natural kinds with discrete causes also worked well for one of the first psychiatric diseases identified: general paresis, known at that time as *general paralysis of the insane*. General paresis, a diverse set of neuropsychiatric symptoms, was described as early as 1822, but its cause was not found until the beginning of the 20th century when syphilitic bacteria were identified in the brains of deceased paretics (Kendler et al., 2010). The model of specific diseases with specific causes was soon generalized to the rest of medicine including psychiatry. In 1912, the German psychiatrist Alfred Roche stated that the “success achieved here has perhaps been a misfortune in its side effects because it nourished the illusion that something similar might soon be repeated” (Sass, 2007; p. 139).

The idea that mental disorders are distinct kinds that behave similarly to other medical conditions has been present throughout the history of psychiatry (Lilienfeld, 2014). For example, Gerald Klerman put forward a summary of fundamental principles of psychiatry in 1978 during his time as chief of the US national mental health agency. Among other less controversial points Klerman suggested that “there is a boundary between the normal and the sick,” and that “there are discrete mental disorders” (Klerman, 1978; for other prototypical examples, see: Guze, 1992; Andreasen, 2001). Especially the rise of biological psychiatry fostered the notion of mental disorders as discrete conditions. Once the DSM-III was established, clinical trials demanded strict diagnostic criteria leading to homogeneous groups of patients, with the aim of developing specific treatments for particular disorders, and of finding specific underlying biological abnormalities (Roth, 2001). Such beliefs in the categorical nature of mental disorders are also reflected in more recent developments like the DSM-5 (Lilienfeld, 2014).

The belief that mental disorders are discrete entities is prevalent among both laypeople and medical professionals (Haslam and Ernst, 2002; Adriaens and De Block, 2013). An implicit essentialist worldview develops early in human cognition (Gelman, 2009) and applies to numerous domains of classification such as chemical elements, species, and emotions (Haslam et al., 2000; Prentice and Miller, 2007); there is a natural human tendency to essentialize. However, categorical systems such as basic emotions or mental disorders may often reflect this *essentialist bias* and not necessarily reality (Allport, 1954; Zachar and Bartlett, 2002).

In depression research, dimensions are transformed into categories by setting threshold values for sum-scores of symptoms which, if exceeded, assign individuals with diverse symptoms to the category of MD. The search for potential causes then often proceeds as if depression is a natural kind, similar to tuberculosis – with disappointing results. We have failed to find *depresso-coccus*, and the quest for biomarkers and more efficacious treatment has been disappointing at best. This lack of progress is partly because the definition of MD as disease entity has encouraged lack of attention to specific symptoms (Persons, 1986; Costello, 1993; Parker, 2005) and their dynamic interactions (Borsboom and Cramer, 2013).

### Depression as Common Cause for its Symptoms

The second assumption tacitly underlying the majority of modern depression research is that depression causes its symptoms, an idea that also goes back to infectious diseases. A measles infection causes measles symptoms, which is why these symptoms are measured to indicate the presence or absence of measles. In the statistical literature, this is referred to as *common cause framework* (Cramer et al., 2010; Schmittmann et al., 2013).

**FIGURE 1:**
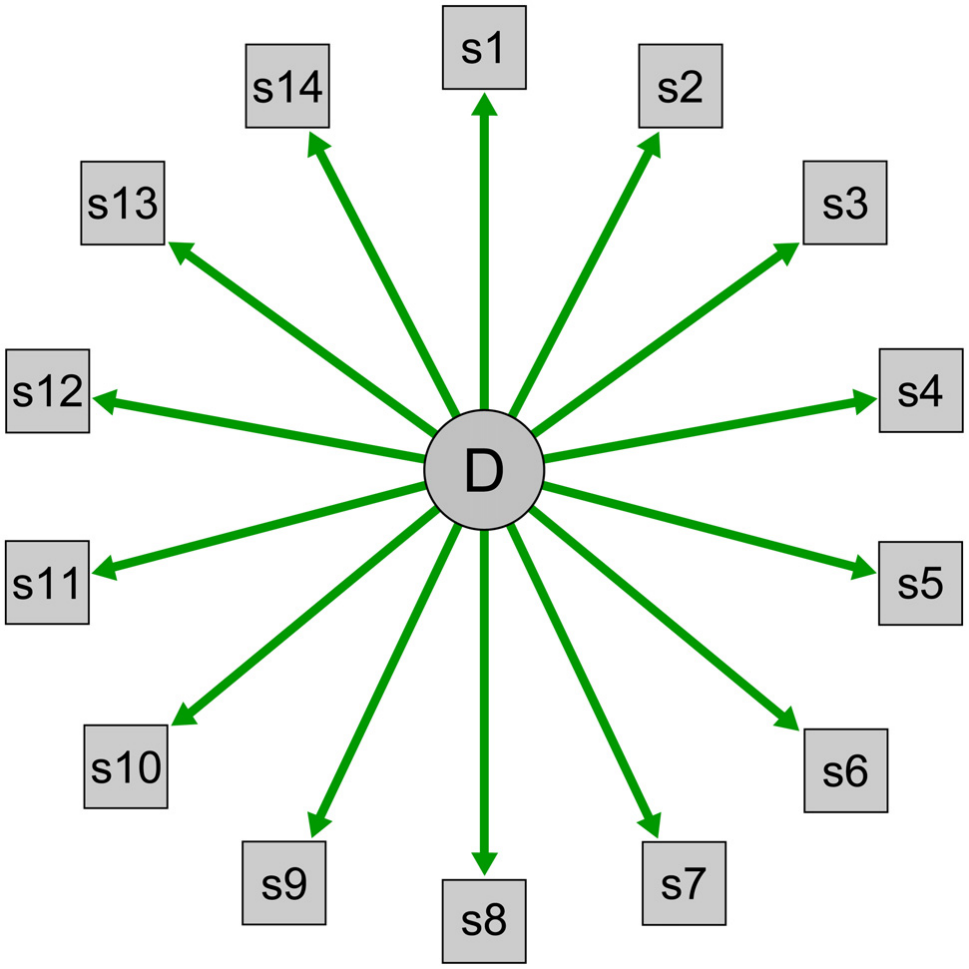
**Visualization of a reflective latent variable model.** D indicates the latent disorder depression that is modeled as common cause of the observable symptoms s1–s14.

Within this framework, underlying concepts are described as reflective latent variables. A latent variable is something that cannot be observed directly; psychological constructs such as intelligence and neuroticism are good examples. A latent variable is *reflective* when the latent variable determines its indicators (Bollen, 1989; Schmittmann et al., 2013; **Figure 1**). The personality trait extraversion, for example, is viewed as the cause of a person’s tendency to enjoy talking to strangers or attending social events; this is why personality tests use these items to measure extraversion. Extraversion is the common cause for extraverted behavior, and we assess such behaviors as indicators of a person’s position on the latent variable extraversion.

Medical disorders are also conceptualized within this framework. When a patient complains about the symptoms polyuria (frequent urination), polydipsia (increased thirst), and polyphagia (increased hunger), a doctor will conclude that the latent disorder diabetes is the most probable common cause for the symptoms. Symptoms are observable indicators that measure an underlying construct. Similarly, we use instruments such as the Beck Depression Inventory (BDI; Beck et al., 1961) or the Hamilton Rating Scale for Depression (HRSD; Hamilton, 1960) to query individuals about depressive symptomatology to investigate the presence or absence of the underlying disease entity, and we model depression via reflective latent variables in structural equation models such as factor analytic techniques. These models describe a clear direction of causation, and arrows always lead from depression to the symptoms in visual representations of latent variable models (**Figure 1**).

This common cause framework renders all symptoms roughly equally central to a disorder, because all symptoms result from an underlying condition; symptoms become diagnostically equivalent and interchangeable (Cramer et al., 2010; Lux and Kendler, 2010; Schmittmann et al., 2013). And while the DSM features are hierarchical structure with two core symptoms of which at least one must be present for a diagnosis, screening instruments do not make this distinction. Overall, sum-scores can be naturally accommodated within this framework of a common cause: symptom number, not symptom nature matters. Consequently, 15 points on the BDI indicate a higher depression severity than 10 points. This is problematic for a variety of reasons: one example is that symptoms differ from each other in their impact on impairment of functioning (Tweed, 1993; Fried and Nesse, 2014).

Another consequence of the common cause framework is that symptoms are considered *locally independent* (Holland and Rosenbaum, 1986; Schmittmann et al., 2013). Depression symptoms tend to cluster, and patients often report a host of associated symptoms such as insomnia, fatigue, appetite loss, sad mood, and concentration problems. In reflective latent variable models, the reason for symptom covariation is the latent variable itself. Imagine we measure a person’s weight (the latent variable) on ten different scales (the tests), and find that the results are highly correlated. In this case, the reason for the high correlations is the latent variable itself, and controlling for the common cause (weight) makes the correlations disappear. The correlations are *spurious*. Likewise, depression symptoms in reflective latent variable models are assumed to be uncorrelated beyond their shared origin. While the common cause framework requires that symptoms be locally independent, every clinician knows that this requirement is implausible: insomnia may cause fatigue, which in turn can trigger concentration problems and psychomotor problems.

The idea of depression as common cause for its symptoms is related to the notion of depression as a distinct disease. Together, these assumptions have fostered a scientific framework in which particular symptom information is ignored in favor of unspecific sum-scores. Over a century after the discovery of biological causes of general paresis, mental disorders such as depression are understood to be natural kinds with essences that fundamentally define them. This explains our quest for biomarkers: if depression is a distinct disease entity, similar to tuberculosis, we ought to find particular biological correlates (e.g., in the brain) that cause depression symptoms. The view is reflected in the recently announced commitment of the NIMH to fund only research examining the neurobiological roots of mental disorders in the current grant cycle (Reardon, 2014).

## Disease Model and Reality: Assumptions Contrast with Evidence

While the majority of depression research is implicitly based on these two assumptions, a host of studies have documented that they do not “fit the data.”

### Depression as Natural Kind

The categorical view of depression as discrete disease is not consistent with taxometric or psychometric data (Kendell and Jablensky, 2003; Aggen et al., 2005; Slade and Andrews, 2005; Ruscio et al., 2007; Markon et al., 2011). Depression symptoms in general population samples do not form non-overlapping distributions for healthy and depressed individuals. This means that there is a lack of a *zone of rarity* – depression is not a discrete category like measles, but a dimension. Research on subthreshold depression supports this view. The presence of fewer than five DSM symptoms is often clinically significant, with depression-like levels of functional impairment, psychiatric and physical comorbidities, and increased risk of future depressive episodes (Pincus et al., 1999; Solomon et al., 2001). While categorical definitions may be necessary for practical purposes, they have fostered reductionist thinking about depression. If we read and talk about depression as one entity, misleading questions such as “what causes it” and “what are genetic predispositions for it” arise, further fortifying essentialist views.

We have also failed to identify pathognomonic biological markers for depression. While the DSM-III (APA, 1980) preamble, written in the spirit of biological psychiatry, predicted that biomarkers reliably associated with most diagnoses would be identified by the time the DSM-IV (APA, 1994) appeared, not a single biological test was ready for inclusion in the DSM-5 over three decades later (Kapur et al., 2012). Large genome-wide association studies have been unable to replicate genetic associations with depression diagnosis (Lewis et al., 2010; Shi et al., 2011; Wray et al., 2012; Daly et al., 2013) or treatment response (Tansey et al., 2012), and in a recent study with over 34000 subjects, no single locus reached genome-wide significance (Hek et al., 2013).

The high comorbidity rates of depression with other disorders such as generalized anxiety disorder (GAD) and posttraumatic stress disorder (PTSD; Kessler et al., 2005) pose another problem for the notion of discrete diseases. Depression is a fuzzy category that substantially overlaps with various other syndromes. Not surprisingly, associations of genetic markers with particular mental disorders are small at best, and often not specific to one diagnosis (Kendler, 2005; Purcell et al., 2009). Moreover, considerable genetic correlations among mental disorders have been identified, for instance between MD and schizophrenia (*r* = 0.43), and between MD and attention-deficit/hyperactivity disorder (*r*= 0.32; Lee et al., 2013). Glutamate neurotransmission provides another excellent example for transdiagnostic similarities, and dysregulations have been implicated in the etiology of depression (Sanacora et al., 2012), schizophrenia (Schwartz et al., 2012), obsessive-compulsive disorder (Grados et al., 2013), and anxiety disorders (Riaza Bermudo-Soriano et al., 2012).

A further argument is that natural kinds are described by properties that are both necessary and sufficient. Depression is measured via rating scales such as the BDI or the HRSD that differ substantially from each other – and from the DSM-5 criteria – regarding the symptoms they assess depression with. This dramatic heterogeneity of symptoms (Olbert et al., 2014; Zimmerman et al., 2014; Fried and Nesse, 2015) is hard to reconcile with the notion of a clearly defined essence of MD.

Finally, biological systems are highly interdependent: genes express proteins that work in cells that ultimately shape behavior – and at most levels, regulatory feedback mechanisms with the environment exist. Declaring one of these processes to be a fundamental part of the essence of a mental disorder is arbitrary and ignores the complex nature and dynamic causality of biological systems (Zachar, 2002; Kendler and Baker, 2007).

Despite all the efforts, major discoveries validating psychiatric disease categories are absent. Due to this lack of validity, critical voices have surfaced calling psychiatry a semi-science (e.g., Brooks, 2013). The president of the American Psychiatric Association, Jeffrey Lieberman, recently responded to such criticism and stated that progress “has been largely limited by technology” (Lieberman, 2013). The human genome and brain are highly complex, and identifying disturbed brain areas, dysfunctional neurotransmitter systems, and risk alleles is a very difficult matter – but ultimately a matter of time and technology. While there is nothing wrong with the idea that psychological problems may have biological correlates – there is some evidence that particular depression symptoms or syndromes are differentially associated with biological markers (Myung et al., 2012; Kendler et al., 2013) – it is noteworthy that the reason for the current lack of progress is generally searched for in technological areas. The disease model itself – our understanding of mental disorders in general and depression in specific – remains largely unquestioned.

### Depression as Common Cause for its Symptoms

There is also compelling evidence showing that the common cause model and its consequences such as symptom equivalence and local independence do not describe depression well. A large number of studies have shown that depression symptoms directly influence each other (e.g., Borsboom and Cramer, 2013; Wichers, 2013; Bringmann et al., 2014). For instance, insomnia can lead to other symptoms such as psychomotor and cognitive impairment, fatigue, low mood, and suicidal ideation (Fawcett et al., 1990; Pilcher and Huffcutt, 1996; Fairclough and Graham, 1999; Durmer and Dinges, 2005; Ferentinos et al., 2009; de Wild-Hartmann et al., 2013), whereas hopelessness is a well-established predictor for suicidal ideation (Beck et al., 1990; Fawcett et al., 1990; Brown et al., 2000; Kuo et al., 2004). The idea that symptoms form vicious circles and influence or maintain each other is also well established in clinical theories (Beck et al., 1979; Ma and Teasdale, 2004), and individuals often describe their own symptoms as dynamic patterns (Frewen et al., 2012, 2013).

Furthermore, symptoms are not equivalent or interchangeable, seeing that they differ in core aspects such as risk factors, precipitants, underlying biology, and impact on impairment (Keller et al., 2007; Kendler et al., 2013; Fried and Nesse, 2014; Fried et al., 2014). Life events like romantic loss or chronic stress lead to different, particular symptom profiles (Keller et al., 2007), and fatigue has different risk factors than, for example, suicidal ideation, making a common cause explanation implausible (Fried et al., 2014). To my knowledge, three studies have aimed to directly test the common cause model and found that alternative models described the data significantly better (Cramer et al., 2013; Fried et al., 2014, 2015).

## Problems Resulting from the Two Assumptions

The tacit adherence to essentialism and the common cause model may be at the very heart of many unsolved problems in depression research. The difficulty is not so much that all clinicians and researchers actively hold this perspective – there are outspoken opponents of the disease model of natural kinds and the common cause framework in psychology, psychiatry, and philosophy (Zachar, 2002; Zachar and Kendler, 2007; Kendler et al., 2010; Cramer et al., 2013; Schmittmann et al., 2013; it is if of note that this critique is aimed at certain assumptions about mental disorders, and thus differs entirely from critics that question the foundations of psychiatry as such (e.g., Szasz, 1974). The problem is that the assumptions are deeply rooted in the history of medicine, are easy to understand and intuitive, usually implicit, and thus reflected in many modern research practices (such as the use of sum-scores). As stated above, the scientific community holds these views implicitly, whereas the majority of individual researchers may not. These assumptions, however, have contributed to a host of problems.

First, they have led us to think simplistically about depression, and ignore important information only an analysis of individual symptoms can provide (Persons, 1986; Costello, 1993; Fried et al., 2014).

Second, the lack of homogeneity of the depressive syndrome may help explaining the low *reliability* of depression diagnosis. The DSM-5 field trials estimated the reliabilities of selected DSM-5 diagnoses in large representative clinical populations (Regier et al., 2013); reliability was assessed by measuring the degree to which two clinicians independently agreed on the presence or absence of psychiatric conditions. The trials yielded a “questionable” reliability of depression diagnosis of 0.28, indicating a very low agreement. The degree of diagnostic certainty was much lower for depression than for the majority of other disorders such as borderline personality disorder (0.54) or PTSD (0.67). David Kupfer, chair of the DSM-5 task force, had to “acknowledge that the relatively low reliability of major depressive disorder […] is a concern for clinical decision-making” (Kupfer, 2013).

Third, there is a lack of *validity* for depression diagnosis, a problem that has received considerable attention in recent years. In a review on the topic, Parker (2005) concluded that depression fails to meet orthodox criteria for validity such as a clear clinical presentation, precise diagnostic boundaries, and treatment specificity. Parker further documented that depression is not a particularly useful label because it does not provide non-trivial information about prognosis and treatment – prediction about the future course of depression is only possible on the level of the individual (e.g., age, gender, neuroticism), but not based on the diagnosis itself. Parker’s views are widely shared. For example, Thomas Insel, director of the NIMH, announced shortly before the release of the DSM-5 that the NIMH would no longer accept the DSM diagnostic criteria as gold standard of psychiatric research due to their lack of validity (Insel, 2013), and the introduction of a leading psychiatric textbook reads: “there is little reason to believe that these categories are valid” (Grebb and Carlsen, 2009). The ‘Research Agenda for DSM-5′ (Kupfer et al., 2002) nicely summarizes different lines of evidence for the lack of validity of diagnostic categories such as the inability to validate them using biological tests, the high comorbidity rates between mental disorders, the high degree of temporal diagnostic instability, and the lack of treatment specificity.

## A symptom-Based Framework for Studying Depression

If depression is not a consistent syndrome, if symptoms differ from each other in important aspects, and if sum-scores obfuscate important information, how should we then understand and model depression? A symptom-based framework offers a viable alternative grounded in scientific realism instead of problematic assumptions. This framework substitutes the two assumptions discussed above – depression as natural kind, and depression as common cause for its symptoms – with two new perspectives.

### Homeostatic Property Clusters

The ontological side of the framework – what is depression? – replaces the notion of depression as bounded category. Instead, depression is understood as a *homeostatic property cluster* (HPC; Boyd, 1991, 1999; Ereshefsky, 2007; Wilson et al., 2007). The idea of HPCs is best introduced using the example of biological species. Assuming that species are discrete natural kinds with clearly demarcated boundaries is pre-Darwinian – today we know that variation and heterogeneity within a species is not a deviation from the true essence of a biological kind, but part of what it is to be a member of those kinds. The reason why genetic, behavioral, and physiological properties of a specific species are contingently clustered in nature is that the presence of one property tends to favor the presence of another. This means that species are property clusters that share related features due to the existence of a multitude of underlying causal mechanisms lawfully connecting these properties. We are inclined to describe HPCs as natural kinds (Zachar and Bartlett, 2002), but since relationships between properties are often probabilistic and not deterministic, imperfect aggregations of properties exist, and most things may show some but not all properties of a property family. The large majority of individuals of any given species are more closely clustered on a multidimensional space of properties than individuals of other species. However, some clusters are closer together than others, and many clusters may overlap partially.

Depression fits the description of a HPC (Kendler et al., 2010; Borsboom and Cramer, 2013). MD is highly heterogeneous, not everybody has all symptoms, and symptoms are associated lawfully with each other due to complex mechanisms. As expected from a property cluster, it is hard to draw a discrete boundary between healthy and ill. The perspective explains the high comorbidity rates of depression with other disorders like GAD and PTSD. Traditional approaches suggest that mental disorders are separate disease kinds, and individuals with comorbid disorders suffer from two distinct conditions. This is explained by a general susceptibility toward negative affect, or by shared genes that predispose for both disorders (Mineka et al., 1998; Barlow et al., 2004). From the perspective of HPCs, it is to be expected that individuals in a property cluster A (e.g., MD) will often be found in another cluster B (e.g., GAD) because the clusters share defining properties. The DSM criteria for MD and GAD both encompass ‘sleep problems,’ ‘fatigue,’ ‘concentration problems,’ and ‘psychomotor agitation,’ and MD and PTSD share the symptoms ‘loss of interest,’ ‘concentration problems,’ ‘sleep problems,’ ‘low mood,’ and ‘self-blame.’ Syndromes substantially overlap, and individuals with a certain set of symptoms will often be described equally well by different diagnoses. This also explains the problems in key research areas such as lack of efficacy of treatment studies, the lack of biological markers for depression, and the low reliability and validity of depression diagnosis: depression is an extraordinary fuzzy syndrome (Fried and Nesse, 2015), and research based on arbitrary thresholds does not properly acknowledge the pronounced heterogeneity of MD and the lawful connections among symptoms.

### Depression as Symptom Network

Moving from the ontological to the statistical side of the framework, the idea of depression as a network of causally related symptoms that interact dynamically provides an alternative to reflective latent variable models. Depression is not understood as a latent disease entity; instead, it is *constituted* by causal connections among symptoms (Borsboom and Cramer, 2013; Schmittmann et al., 2013; van de Leemput et al., 2014). In other words, symptoms do not cluster because of a shared origin – they cluster because they trigger each other. This perspective naturally accommodates findings such as differences among symptoms in their risk factors and underlying biology, and networks do not presuppose symptoms to be interchangeable or equivalent. The framework also focuses on the causal autonomy of symptoms instead of assuming that they are passive products of a common cause: insomnia may lead to fatigue, which in turn can cause concentration and psychomotor problems. Although a review of the network literature is beyond the scope of this report (see Borsboom and Cramer, 2013), I will list a number of recent illustrative network studies that have addressed important research problems in different domains.

First, the framework is useful in comorbidity research, utilizing evidence that symptoms trigger other symptoms *irrespective* of a given diagnosis. For instance, Cramer et al. (2010) have shown that MD and GAD constitute two related psychopathological clusters that overlap considerably, which can be explained by bridge symptoms such as ‘insomnia’ that connect the clusters. Related work has shown that the DSM symptom network conforms to what can be called a *small world structure*: the DSM features a host of interrelated symptoms, and symptoms are strongly connected both within and across diagnoses. This means that one can “travel” from any symptom to any other symptom in just a few jumps (Borsboom et al., 2011; Goekoop and Goekoop, 2014), a perspective that offers new possibilities for comorbidity research.

Second, there is evidence that symptom networks vary as a function of the type of life event experienced recently (Cramer et al., 2013; Fried et al., 2015). For instance, the association between the symptoms depressed mood and thoughts of death was stronger in participants after a social conflict compared to a group who had experienced a romantic loss. This is consistent with previous work documenting that particular life events are associated with specific symptom profiles (Keller et al., 2007), and contrasts with the idea that all depression symptoms are products of one shared origin (and – in turn – with the notion that the treatment of such a common cause will relieve all depression symptoms).

Third, the network perspective allows for mutually reinforcing symptoms and feedback cycles, a notion that has long been acknowledged by clinicians (Beck et al., 1979; Ma and Teasdale, 2004). For example, worthlessness and guilt can form a vicious circle in depressed patients, leading to a situation that is self-sustaining and hard to escape (Bringmann et al., 2013). Such reciprocal interactions are common in empirical psychopathological networks, but difficult to estimate in traditional structural equation models that usually presuppose local independence among symptoms (i.e., that symptoms are uncorrelated beyond their common cause; see Schmittmann et al., 2013).

Fourth, network models can help identify the most causally central symptoms – symptoms that trigger others across time – which has important implications for prevention and intervention research. A central symptom is one that is connected to many other symptoms in the network, whereas a peripheral symptom features few or no connections. In a recent paper, Robinaugh et al. (2014) used a network analysis to establish that symptoms of persistent complex bereavement disorder (PCBD) form a syndrome that is related to, but somewhat distinct from a network of depression symptoms. The symptom ‘emotional pain’ had the highest centrality among individuals suffering from PCBD. This means that endorsing emotional pain likely leads to the activation of subsequent symptoms, and that the symptom deserves special attention in the context of PCBD. Network-based centrality metrics (see Opsahl et al., 2010) thus offer insights different from the investigation of symptom severity (there is not necessarily a relation between how severe a symptom is and how central it is in a given network).

Fifth, novel statistical methods that closely follow participants over time allow for constructing personal psychopathological networks for each participant (Bringmann et al., 2013); this means that the network framework allows for nomothetic analyses on the group level as well as idiographic insights on the person level. Person-centered networks may differ across individuals despite the same diagnosis, opening up a range of exciting research opportunities. For instance, Kramer et al. (2014) recently demonstrated that feedback on personalized patterns of affect significantly reduces depressive symptoms compared to a control group without feedback.

Sixth, the network framework may offer insights into the problem of missing heritability: while depression seems to be at least moderately heritable, the identification of specific genetic markers has been difficult (Zuk et al., 2012). As recently documented for symptoms of psychosis (Smeets et al., 2014), certain polymorphisms make individuals more vulnerable to develop particular psychopathological symptoms in response to others. For depression, there may be substantial variability in the way people respond with fatigue to insomnia, or with suicidal ideation to sad mood, because such pathways are likely moderated by the genetic architecture. It is possible that large parts of the missing heritability are hidden in such symptom links, and future genetic studies should examine individual symptoms as well as their associations.

Finally, networks may offer a novel perspective on recurrence and relapse of MD. There is evidence that not only the number of symptoms predicts relapse, but that particular symptoms play an especially important role. Residual anxiety and sleep problems independently predicted earlier MD recurrence in patients who had previously remitted from depression (Dombrovski et al., 2007), and understanding the causal mechanisms that underlie residual symptoms may allow for developing prevention strategies that specifically target populations at high risk for recurrence.

**FIGURE 2:**
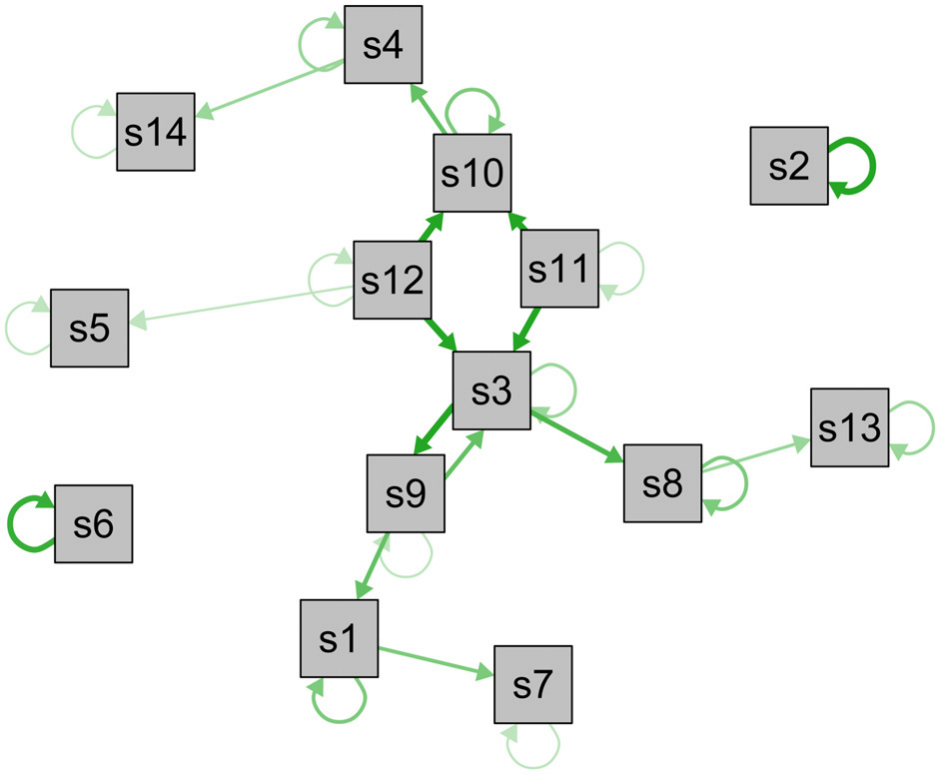
**Visualization of a longitudinal psychopathological symptom network.** Longitudinal network model of the directed associations between depression symptoms s1–s14 (not based on data). Self-loops represent autoregressive effects, edges represent associations among symptoms across time, and line-thickness indicates strength of the associations. Symptoms with a large number of connections are displayed in the center, symptoms with few or weak connections in the periphery.

In summary, understanding depression symptoms as distinct entities organized in webs of direct causal influences may facilitate progress in a large number of key research domains. This is the case for both the group level as well as for the study of idiographic aspects of depression. To contrast the common cause model visualized in **Figure 1**, I constructed a longitudinal symptom network to illustrate how such a network can look like (**Figure 2**; not based on data; for empirical networks, see Bringmann et al., 2014; Pe et al., 2014; Robinaugh et al., 2014). In this exemplary network, especially symptoms s3, s11, and s12 are closely interconnected and central – an intervention here would likely stop the activation of subsequent symptoms. Other symptoms such as s2, s5, or s6, however, have few or no connections and may thus not be of great clinical importance. Furthermore, s3 and s9 have reciprocal effects resembling a vicious circle, and s2 and s6 have particularly strong autoregressive effects (self-loops), implying that the presence of these symptoms in the near future can be reliable predicted by their presence at the current timepoint.

Two limitations of current network studies should also be discussed briefly. First, while the majority of prior research has focused on nomothetic aspects of networks, the degree of heterogeneity across participants remains largely unclear. For example, feeling worthless may trigger feeling guilty in only half of a study sample, whereas the opposite may be the case for the rest of the participants; averaged group-level networks may not properly reflect such differences (Rosmalen et al., 2012). An important step forward is to examine the heterogeneity of networks across participants, which is possible with statistical tools like the multilevel vector autoregression model for experience sampling data (Bringmann et al., 2013) that allow to construct idiographic networks and can derive measures of heterogeneity on the group level. A related step is the development of mixture models that detect subgroups of individuals with homogeneous networks.

A second challenge is the investigation of the statistical fit of networks. Absolute and relative fit indices that are well established in other fields (for an overview, see Hooper et al., 2008) are not yet routinely implemented to determine the fit of network models. This makes it difficult to examine how well a network describes data, or to statistically compare different networks (e.g., two groups of healthy and depressed participants).

Due to the growing popularity of networks in the psychological literature in recent years, they have become an active field of development both in clinical-substantive (e.g., Hofmann, 2014) as well as in the theoretical-psychometric research domains (e.g., van Borkulo et al., 2014). Ultimately, the question whether the network framework can be considered a successful enterprise will depend on the insights it provides as a model for depression. If the centrality of a given MD symptom, the density of a depression network, or the way specific emotions shape others over time allow us to predict important clinical variables such as the increase in depressive symptomatology, treatment outcome, or relapse, the notion of depression as a dynamical system deserves a place among other more established theories.

## Conclusion

While psychiatric diagnoses are necessary for standardizing research and treatment, the last decades have not brought substantial progress toward validating diagnostic categories, and identifying clearly demarcated boundaries between diseases remains difficult. In this paper I suggest a solution that consists of two steps. First, it is important to acknowledge that many research practices are based on two problematic assumptions – that depression is a discrete disease category, and that depression causes its symptoms. A second step forward is to identify a suitable alternative disease model that is based on more realistic assumptions, and I believe that the network approach may be a good candidate.

Depression is not a natural kind – it is a fuzzy and heterogeneous disease. But the most important feature of a diagnosis is not that it exists outside human classification systems as real entity (Fine, 1984); above all, a diagnosis should be *useful* (Zachar, 2002; Parker, 2005; Kendler et al., 2010). And a diagnosis is useful if it provides clinical utility, as suggested by the DSM (APA, 2013; p.20): “it should help clinicians to determine prognosis, treatment plans, and potential treatment outcomes for their patients.” In other words, a diagnosis should make predictions about etiology, disease onset, course of illness, and recurrence, and thus allow for the development of efficacious prevention and treatment strategies. Important features of a useful diagnosis are a small number of symptoms specific to the disease (symptomatic homogeneity) along with a few strong risk factors (etiological homogeneity). A disorder with a homogeneous pool of pathognomonic symptoms allows for a reliable diagnosis. It increases the probability to discover distinct pathophysiological processes, and enables researchers and clinicians to develop and implement more specific and well-directed treatment strategies. A disorder with a clearly circumscribed etiology, on the other hand, makes early detection and prevention easier. Depression is highly heterogeneous on both dimensions: it is associated with a large number of symptoms, and countless pathways can lead to an episode of MD. If we additionally consider the low reliability, low validity, and high comorbidity rates of depression, it is fair to ask whether MD is a particularly useful diagnosis.

Adopting a novel network research framework may allow for substantial progress. This network approach focuses on smaller and more valid and reliable units of observation – symptoms – that are likely associated with more distinct underlying pathophysiological processes. The framework not only acknowledges the highly heterogeneous nature of MD, its complexity, and its fuzzy boundaries, it also puts the focus on the dynamic interactions among symptoms that have received comparably little attention. The investigation of the differences between symptoms in terms of risk factors or biomarkers, as well as their causal pathways may be a great opportunity. In addition, future network studies could reveal that variables such as life stress, personality traits, and pathophysiology moderate specific symptom pathways: some individuals may be especially vulnerable to develop fatigue in response to insomnia, depending on their life situation or genetic background.

Ultimately, insights gained through this symptom-based approach may enable us to better describe several more valid and reliable depressions as related property clusters of associated symptoms in the DSM-6. The network framework thus offers novel perspectives for constructing an empirically derived classification system in which psychological and biological perspectives are no longer competing, but complementing aspects.

## Conflict of Interest Statement

The author declares that the research was conducted in the absence of any commercial or financial relationships that could be construed as a potential conflict of interest.
